# Pure ICL Implantation: A Novel Ophthalmic Viscosurgical Device-Free Method

**DOI:** 10.1155/2021/7363267

**Published:** 2021-10-06

**Authors:** Qin Qin, Lianyun Bao, Zifang He, Feifei Chen, Dandan Zhu, Si Zhang, Wenwen Zhang, Yajun Liu, Ruiying Gao, Zhenggao Xie

**Affiliations:** ^1^Nanjing Drum Tower Hospital, The Affiliated Hospital of Nanjing University Medical School, Nanjing 210008, Jiangsu, China; ^2^Nanjing Drum Tower Hospital Clinical College of Nanjing Medical University, Nanjing 210008, Jiangsu, China; ^3^Nanjing Drum Tower Hospital Clinical College of Traditional Chinese and Western Medicine, Nanjing University of Chinese Medicine, Nanjing 210008, Jiangsu, China

## Abstract

**Purpose:**

To assess the clinical efficiency of a novel ophthalmic viscosurgical device-free (OVD-free) method for intraocular collamer lens (EVO-ICL) implantation in myopic eyes.

**Methods:**

In this study, 40 patients underwent ICL implantation for both eyes: one eye underwent traditional ICL implantation, and the other eye underwent OVD-free (pure) ICL implantation. Preoperative and postoperative UDVA, BCVA, equivalent spherical degree (SE), IOP, visual quality index, subjective visual quality scale, corneal endothelial cell density (ECD), operation time, and complications were compared between and within the traditional and pure ICL implantation groups.

**Results:**

Increased IOP >22 mmHg 2 h after surgery was noted in 8 eyes (20%) in the traditional group, but not in the pure group (0%, *P* < 0.001). Increased IOP relative to baseline was significantly higher at 2 h after surgery for the traditional group compared with the pure group (*P* < 0.001). UDVA, BCVA, and SE were significantly improved in the pure group compared with those in the traditional group 1 day (*P* < 0.001, *P*=0.003) after implantation, but not 1 week or 3 months after. Modulation transfer function cut-off frequency (MTF cut-off), Strehl ratio (SR), and OV20% were significantly better in the pure group than in the traditional group 1 day after implantation (*P*=0.013, *P*=0.009, and *P*=0.004). No significant difference in ECD changes within or between groups was observed (*P* > 0.05). The operation time for the pure group (2.897 ± 0.346 min) was significantly shorter than that for the traditional group (4.444 ± 0.656 min; *P* < 0.001). No complications were reported for either group during the observation period, except early IOP elevation in the traditional group.

**Conclusions:**

The pure ICL implantation method was associated with faster visual acuity recovery, shorter operation time, and more stable intraocular pressure. Pure ICL represents a safe and convenient method for ICL implantation compared with the traditional method, completely eliminating OVD-related complications without causing additional complications.

## 1. Introduction

Myopia describes the condition in which the refractive system of the human eye focuses parallel light in front of the retina in a relaxed state [1]. In China, approximately 700 million people suffer from myopia, and 10% of those suffer from high myopia. Various surgical procedures can be used to correct myopia, including posterior chamber phakic intraocular lens (PIOL) implantation, small incision lenticule extraction (SMILE), and laser-assisted in situ keratomileusis (LASIK); however, the surgical options for high myopia patients with limited corneal thickness remain limited to lens replacement and PIOL implantation.

Visian ICL™ (STAAR Surgical, Nidau, Switzerland) is a type of posterior chamber PIOL [2, 3], and previous studies have shown that EVO-ICL (implantable collamer lens with a centre hole) implantation is satisfactorily safe [4]. EVO-ICL implantation can reduce the induction of high-order aberrations and improve contrast sensitivity [5]. Because the intraocular position of EVO-ICL is close to the eyeball node, magnification approaches 1, and the imaging size of external objects on the retina is similar to that for emmetropia, resulting in EVO-ICL implantation patients presenting better visual quality than patients who undergo corneal refractive surgery. Features such as good postoperative visual acuity, visual quality, and reversibility have made EVO-ICL the first-line choice for an increasing number of patients with high myopia [6–12].

Traditional ICL implantation requires ophthalmic viscosurgical device (OVD) injection into the ICL implant container and the anterior chamber (AC) to maintain the operating space and protect the corneal endothelium and lens. Failure to completely clear OVD postoperatively can result in an acute increase in intraocular pressure (IOP) at the early postoperative stage [13–16]. Intraoperative OVD use may also increase the costs and time required for surgery [17]. Prolonged surgery and OVD irrigation using a syringe-coupled injection needle may increase the likelihood of contact between the ICL and the transparent lens, leading to early lens opacity after operation [18, 19]. The viscosity and electrostatic charge of OVD materials may result in the suspension of inflammatory cells in the AC, increasing the probability of postoperative inflammatory reactions. To address the adverse effects associated with OVD use, we developed a technology that uses a continuous infusion of balanced salt solution (BSS) through a side port, completely eliminating the need for OVDs during ICL implantation. We named this method “pure ICL implantation.” A preprint has previously been published [20]. In the present study, the double-pass technique (OQAS™ II, Optical Analysis System, Visiometrics, Spain) was used to assess optical quality indicators and intraocular objective scattering index (OSI) in patients who underwent either pure or traditional ICL implantation. The National Eye Institute Refractive Error Quality of Life Instrument-42 (NEI-RQL-42) score was used to evaluate the subjective visual perceptions of the patients. The intraocular pressure (IOP), uncorrected distance visual acuity (UDVA), best-corrected distance visual acuity (BCVA), equivalent spherical degree (SE), corneal endothelial cell density (ECD), operation time, and complications were also examined. The results of this study provided additional information regarding the safety and effectiveness of pure ICL implantation, providing a basis for the clinical popularisation of this method.

## 2. Patients and Methods

### 2.1. Patients

From 2018 to 2020, a total of 80 eyes in 40 patients (18 men and 22 women) underwent EVO-ICL implantation at Nanjing Drum Tower Hospital, affiliated with Nanjing University Medical School. One eye of every patient underwent the pure method, and the other underwent the traditional method. Patients with a history of eye surgery, severe dry eye, progressive corneal degeneration, cataracts, retinal detachment, or uveitis were excluded. The Institutional Review Board at Nanjing Drum Tower Hospital approved this study, which was performed in accordance with the tenets of the Declaration of Helsinki. All patients signed an informed consent concerning the risk of surgery.

### 2.2. Observation and Detection Indicators

Routine preoperative examinations were performed for all eyes, including UDVA, BCVA, scotopic pupil size, anterior segment examination under a slit lamp microscope, and retina examination, using three-mirror contact lenses. IOP was measured with a noncontact tonometer (Topcon Company, Japan). Optometry was performed using a CV-3000 comprehensive optometry testing machine (Topcon Company, Japan), including cycloplegic refraction and subjective optometry. The AC depth and corneal curvature were measured using an OCULYZER II (WaveLight, Alcon). A panoramic ultrasound biomicroscope (UBM) was used to study the anterior segment structures of the eye and measure the sulcus-to-sulcus (STS) distance, which was used to determine the ICL size. We calculated the ideal ICL size as STS +0.7 mm. Modulation transfer frequency (MTF), Strehl ratio (SR), three Optical Quality Analysis System (OQAS) contrast values (OV100%, OV20%, and OV9%), and objective scattering index (OSI) were detected by OQAS II. The ECD was measured using an SP-3000P corneal endothelial cell counter (Topcon Company, Japan). National Eye Institute Refractive Error Quality of Life Instrument-42 (NEI-RQL-42) scores were collected before and 3 months after surgery. The time of each surgery was recorded. Before the operation, 0.5% levofloxacin eye drops (Santen, Japan) were administered for 3 days to prevent infection. 0.1% fluorometholone eye drops (Santen, Japan) and 0.5% levofloxacin eye drops were administered 4 times a day for 2 weeks after the operation. Pranoprofen eye drops were also administered 3 times a day for 1 month after operation. In this study, the same doctor was in charge of all surgeries.

### 2.3. Anterior Chamber Maintainer (ACM)

**Figure 1:**
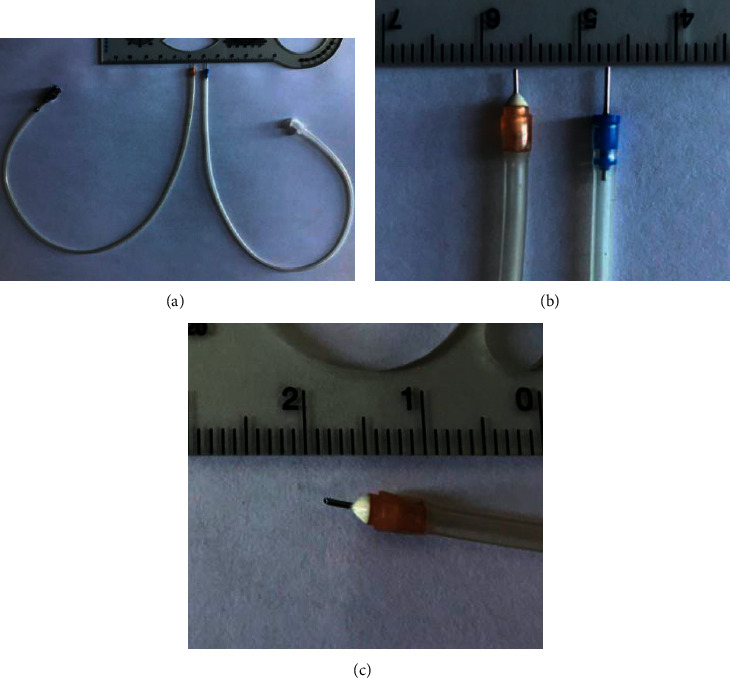
Anterior chamber maintainer. (a) Left one is the ACM; right one is a 25g tube. (b) The length is 1.5 mm. (c) The head is bevelled at a small angle (60°).

The anterior chamber maintainer (ACM), with a 0.4 mm diameter and 1.5 mm length, was designed by our group and produced by an ophthalmic instrument company. The head was bevelled at a small angle (60°) to facilitate access to the AC through the side incision (Figure 1).

### 2.4. Surgical Procedures

#### 2.4.1. Traditional ICL Implantation

Before surgery, the eyes were instilled with compound tropicamide eye drops (Santen, Japan) 4 times to expand the pupil diameter to greater than 7 mm. Oxybuprocaine hydrochloride (Santen, Japan) eye drops were applied 4 times as topical anaesthesia. A small OVD amount (1.7% sodium hyaluronate, Shandong Bausch and Lomb Freda Company) and balanced salt solution (BSS, Alcon, USA) were injected into the ICL implant container. The injector was properly installed and placed in BSS for later use. An assisted right-side incision was made, through which an appropriate OVD amount was injected into the AC. A 3.0 mm clear corneal main incision was made, typically placed on the steep axis of the corneal curvature, to release corneal astigmatism. The ICL was inserted from the main incision of the AC, and a sufficient OVD amount was injected into the front surface of the ICL to maintain the operating space. After gently tucking the footplate beneath the iris and adjusting the ICL to a central position, the OVD was manually irrigated from the AC using BSS by an injection needle attached to a syringe. Finally, the incisions were made watertight.

#### 2.4.2. Pure ICL Implantation

Before surgery, dilated pupil and surface anaesthesia were the same as the traditional methods. The ICL implant container was filled only with BSS, and the ICL injector was installed properly and placed in BSS for later use. Symmetrical assisted incisions were made on the right and left sides, approximately 0.4 mm wide, and the ACM was placed in the left incision. A 3.0 mm transparent corneal main incision was performed, typically located on the steep axis of the corneal curvature, to release corneal astigmatism. The ICL was inserted from the main incision into the AC, gently tucking the footplates beneath the iris and adjusting the ICL to a central position. Finally, the incisions were made watertight.

### 2.5. Management of Early Acute IOP Elevation

Early IOP elevation was defined as an increase in IOP >22 mmHg within 2 h after surgery. If early IOP elevation was detected, a slit lamp examination was performed to eliminate pupil block or malignant glaucoma caused by excessive vault. If IOP was only slightly elevated (IOP <25 mmHg), close observation was performed. For moderately elevated IOP (IOP = 25–30 mmHg), topical antiglaucoma medications were administered. For significantly elevated IOP (IOP >30 mmHg), AC drainage was performed once through the side incision, with or without the administration of topical antiglaucoma medication, to reduce IOP to 10–13 mmHg. After treatment, IOP was measured every 2 h until a normal value was achieved.

### 2.6. Statistical Analysis

Collected data were entered into Microsoft Excel (Microsoft Co. WA, USA) and analysed using Statistical Package for the Social Sciences (SPSS, version 20; IBM Inc., Armonk, USA). Descriptive and inferential statistical analyses were performed. At the descriptive level, continuous parameters were evaluated for parameter normality and are reported as the mean ± traditional deviation (SD). At the inferential level, an independent *t*-test, a paired-sample *t*-test, the Mann–Whitney *U* test, and the one-way analysis of variance (ANOVA) were performed. A *P* value of less than 0.05 was considered significant.

## 3. Results

### 3.1. General Data

No significant differences in age, gender, preoperative SE, UDVA, or BCVA were identified between the two treatment groups (Table 1).

### 3.2. Comparison of IOP between the Two Groups

The average IOP of the pure group is 13.730 ± 2.172, 15.550 ± 2.438, 14.150 ± 2.070, 13.880 ± 2.267, and 13.630 ± 2.618 before operation and 2 h, 1 day, 1 week, and 3 months after operation, respectively, compared with 13.550 ± 2.342, 20.575 ± 2.890, 14.275 ± 1.948, 13.780 ± 2.304, and 13.380 ± 2.906 in the traditional group. Preoperative IOP was within the normal range for both groups with no significant difference (*P*=0.730). A significant difference in the time course of IOP changes was observed between the two groups. At 2 h after operation, no patients in the pure group (0%) had IOP >22 mmHg compared with 8 patients in the traditional group (20%). One patient in the traditional group experienced mild eye pain and corneal oedema, with an IOP of 31 mmHg 2 h after operation. After performing AC drainage and administering carteolol hydrochloride drops twice, the symptoms disappeared within 24 h, and IOP decreased to 18 mmHg. Higher IOP values of the other 7 patients are 23, 24, 24, 23, 26, 25, 23 mmHg. Two of them (25, 26 mmHg) were given carteolol hydrochloride 2 times. One day after operation, both groups presented IOP <22 mmHg.

**Figure 2:**
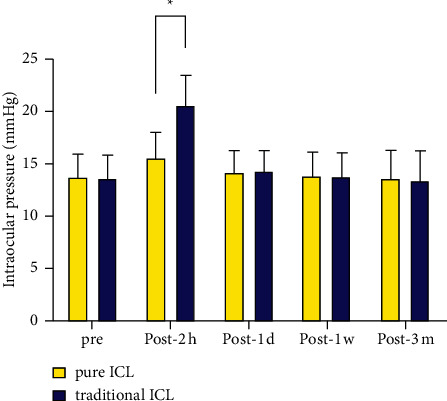
Time course of IOP changes in eyes undergoing pure ICL implantation and traditional ICL implantation. ^*∗*^*P* < 0.001.

At 2 h after surgery, the IOP values in the pure group were significantly lower than those in the traditional group (*P* < 0.001). At 1 day, 1 week, and 3 months after the operation, no significant differences in IOP were observed between groups (*P*=0.782, 0.845, 0.687; Figure 2).

**Figure 3:**
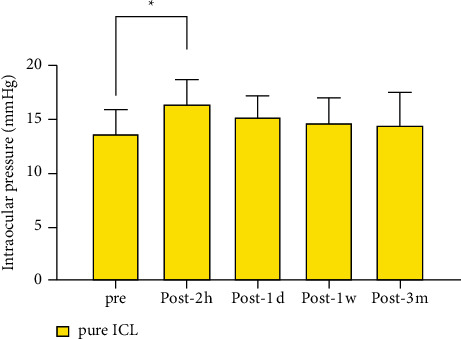
Time course of IOP changes in eyes undergoing pure ICL implantation.^*∗*^*P* < 0.001.

**Figure 4:**
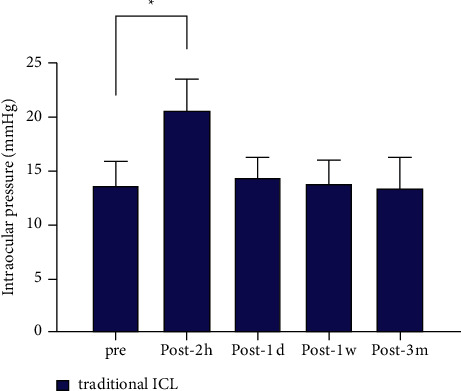
Time course of IOP changes in eyes undergoing traditional ICL implantation. ^*∗*^*P* < 0.001.

A significant difference in IOP was observed before and 2 h after operation in both groups (*P* < 0.001), but no significant differences were observed between other time points (Figures 3 and 4).

**Figure 5:**
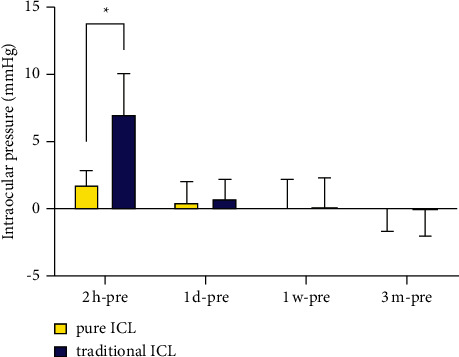
The difference of intraocular pressure between baseline (preoperation) and each postoperation time point in eyes undergoing the pure ICL implantation and traditional ICL implantation. Note. 2 hours postoperatively: *P* < 0.001, 1 day postoperatively: *P*=0.369, 1 week postoperatively: *P*=0.868, 3 months postoperatively: *P*=0.845, ^*∗*^the difference is statistically significant.

The differences between postoperative and preoperative baseline IOP values in the pure group were 1.83 ± 0.96, 0.43 ± 1.55, 0.15 ± 2.02, and −0.1 ± 1.58 at 2 h, 1 day, 1 week, and 3 months after surgery, respectively, compared with 7.03 ± 2.99, 0.73 ± 1.41, 0.23 ± 2.01, and −0.18 ± 1.84 in the traditional group. The differences between baseline and 2 h postoperative IOP values were significantly lower in the pure group than in the traditional group (*P* < 0.001). At 1 day, 1 week, and 3 months after surgery, no significant differences were observed between groups (*P*=0.369, 0.868, 0.845; Figure 5).

### 3.3. Comparison of Visual Acuity and Refractive Power between the Pure and Traditional ICL Implantation Groups

Table 2 shows the visual acuity and refractive power values of both groups. The intragroup comparisons showed increased UDVA and BCVA values for both groups 3 months after surgery compared with preoperative values. Postoperative SE values were significantly lower than preoperative values (*P* < 0.001).

At 1 day after surgery, the UDVA and BCVA values of the pure group were significantly better than those of the traditional group (*P* < 0.001). No significant differences between groups were observed before and 1 week and 3 months after surgery.

On postoperative day 1, the SE of the pure group was significantly lower than that of the traditional group (*P*=0.003). No significant difference in SE was observed between groups at the other time points.

### 3.4. Comparison of Visual Quality between Pure and Traditional ICL Implantation Groups

Table 3 shows the OQAS II visual quality indicators for patients in the pure group before surgery and 1 day, 1 week, and 3 months after surgery. The MTF cut-off frequencies at 1 day and 1 week after surgery were significantly lower than those before surgery (*P* < 0.001 and *P*=0.014). The SR value 1 day after surgery was significantly lower than that before surgery (*P*=0.025). The OV100% values 1 day and 1 week after surgery (*P* < 0.001 and *P*=0.002) and the values of OV20% and OV9% 1 day after surgery (*P* < 0.001 and *P* < 0.001) were significantly lower than the corresponding preoperative values. No significant differences were observed in other OQAS II visual quality indicators between preoperative and postoperative time points. The OSI values 1 day and 1 week after surgery were higher than those before surgery (*P* < 0.001), but no significant difference in OSI values was observed 3 months after surgery and before surgery.

Table 4 shows the OQAS II visual quality indicators for patients in the traditional group before surgery and 1 day, 1 week, and 3 months after surgery. The MTF cut-off frequency, SR, OV100%, OV20%, and OV9% values 1 day (*P* < 0.001, *P* < 0.001, *P* < 0.001, *P* < 0.001, and *P* < 0.001) and 1 week (*P*=0.005, *P* < 0.001, *P*=0.003, *P* < 0.001, and *P* < 0.001) after surgery were lower than the corresponding preoperative values. No significant differences in any OQAS II visual quality indicators were observed before and 3 months after surgery. The OSI values 1 day and 1 week after surgery were significantly higher than that before surgery (*P* < 0.001, *P*=0.003, respectively), but no significant difference in OSI values was observed before and 3 months after surgery.

Table 5 compares the visual quality indicators of patients undergoing the two surgical methods. We compared visual quality between the two groups before and 1 day, 1 week, and 3 months after surgery. At 1 day after surgery, the MTF cut-off frequency, SR, and OV20% values of the pure group were significantly higher than those of the traditional group (*P*=0.013, *P*=0.009, and *P*=0.004). One week after surgery, the SR, OV20%, and OV9% values of the pure group were significantly higher than those in the traditional group (*P*=0.003, *P*=0.047, and *P*=0.002). The remaining values did not differ significantly at any time point.

### 3.5. NEI-RQL-42 Scores

Patients filled out the NEI-RQL-42 before and 3 months after surgery and scored as described by the instructions: the average value of each item = the sum of all points per item/the number of questions per item.

Table 6 shows the within-group comparisons in NEI-RQL-42 scores before and 3 months after surgery. At 3 months after surgery, except for a decrease in glare and no significant increase in near vision, all indicators were significantly higher than those before surgery (*P* < 0.05) for both groups.

Table 7 shows the between-group comparison in NEI-RQL-42 scores before and 3 months after surgery. No significant differences in scores were observed between the two groups.

### 3.6. Comparison of Corneal ECD before and after Operation within and between the Pure and Traditional ICL Implantation Groups

Table 8 shows no significant differences in corneal ECD between the pure and traditional groups either before or three months after surgery (*P* > 0.05). No significant differences were observed between these two time points within each group (*P* > 0.05).

### 3.7. Operation Time and Complications

**Figure 6:**
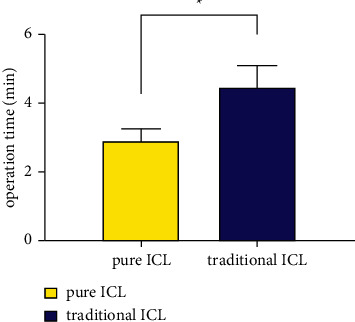
Comparison of operation time between the two groups. ^*∗*^The difference is statistically significant.

The mean operation time for the pure group was 2.897 ± 0.346 min, which was significantly shorter than the operation time of 4.444 ± 0.656 min for the traditional group (*P* < 0.001, Figure 6).

The vault measurements for all postoperative eyes in both groups were between 1/2 corneal thickness (CT) and 3/2 CT at each time point after surgery. Except for early postoperative IOP elevation in some patients, no serious complications, such as posterior corneal elastic layer detachment, cataract development, pupil block, iris injury, or pigment dispersion, occurred during the intraoperative or 3-month follow-up periods.

## 4. Discussion

Through the continuous development and improvement in ICL implantation technology, the EVO-ICL implant with a central hole has become the most mainstream surgical method for intraocular refractive surgery, favoured by patients with high myopia and other refractive errors who are not satisfied with or do not qualify for corneal laser surgery [21]. The safety and efficacy of ICL implantation have been widely recognised [3, 12]. During traditional ICL implantation procedures, OVDs should be injected into the ICL implant container and the eyes to reduce the potential for damage to the corneal endothelium and transparent lens and to maintain the AC space for surgery. However, residual OVDs have been associated with increased or abnormal IOP, lens opacity, and slow vision recovery [13–16, 18, 19]. Bluementhal [22], Tak [23], Fatih [24], Bardoloi [25], and other researchers have used various ACM devices to avoid the use of OVDs during phacoemulsification cataract extraction or intraocular lens implantation. Pan [26] introduced ICL implantation with continuous infusion and a single-handed ACM. Its safety and effectiveness have been confirmed. Pan is the only study to date that used ACM for ICL implantation. However, the use of Pan's ACM requires one hand, which limits the coordination of activities that require two hands, such as eye movement. Therefore, we developed a hands-free, operationally convenient ACM. Under continuous infusion conditions, the ICL implantation could be performed without the use of OVDs, referred to as pure ICL implantation. This method reduces the operation time, promotes the early recovery of vision, and is beneficial for controlling increases in IOP and reducing complications associated with residual OVDs. Pure ICL implantation was found to be effective and worthy of clinical application.

During traditional ICL implantation, the use of OVDs is essential. OVDs function to fill, protect, and lubricate the AC [27, 28]. In this study, the pure ICL implantation method used a continuous BSS infusion through a side port, to eliminate the use of OVDs. Therefore, the ICL spreads faster and does not contact the lens or corneal endothelium, and the flushing process applied to remove OVDs is no longer necessary, resulting in a shortened operation time to approximately 3 min, which was significantly shorter than the time necessary for the traditional method, reducing the risk of corneal endothelial damage associated with prolonged operation times, reducing material costs, and improving the experience of both patients and doctors.

A large number of studies [5, 14–16, 21, 29] have shown that early postoperative IOP elevation is a common complication due to residual OVDs following ICL implantation using traditional methods. The intraoperative use of various types of OVDs may also affect IOP recovery. Gonzalez-Lopez [14] implanted V4c ICL in 100 eyes, among which 5 eyes experienced increased IOP 3–6 hours after surgery. They hypothesised that residual OVDs (2% hydroxyl propyl methyl cellulose) blocked the trabecular meshwork or the central hole of the ICL, resulting in increased IOP. Almalki [15] found that, out of 534 patients after ICL implantation, 58 patients experienced IOP elevation, including 23 patients with IOP elevation on the first day after surgery (the OVD was 1% sodium hyaluronate). Senthil [16] found that, in patients with high IOP after ICL implantation, 27% of cases were caused by OVD residues (2% hydroxyl propyl methyl cellulose), including 2 cases with pupil block due to OVD residue. Our study showed that 2 h after surgery more patients (20%) in the traditional ICL implantation group had IOP >22 mmHg compared with the pure ICL implantation group (0%). The IOP of the traditional group was similar to previous studies.

The differences between the 2 h IOP values and the preoperative baseline IOP values were significantly lower in the pure ICL implantation group than those in the traditional ICL implantation group. The IOP values for both groups returned to normal levels at 1 day, 1 week, and 3 months after surgery, with no significant differences between groups or compared with their preoperative baseline IOP values. The results showed that avoiding OVD use was beneficial for controlling early postoperative IOP elevation and promoting the recovery of IOP after surgery.

The evaluation of visual quality after refractive surgery typically includes both objective and subjective detection methods, such as the objective visual quality indicators measured by OQASII, the intraocular OSI, and the subjective visual quality scale reported by patients [30–32]. In this study, the OQAS™ II double-pass retinal imaging technology was used to analyse the visual quality of the target by collecting data from the 780 nm laser diode light intensity distribution on retinal imaging. The point light source resolution of an optical system is described by the point spread function (PSF). During the analysis of optical imaging quality, the visual quality indicators, including the MTF cut-off frequency, SR, and OVs at various contrast sensitivities (OV9%, OV20%, and OV100%), and the OSI are derived from the PSF. The results of this study showed that the MTF, SR, OV100%, and OV20% values of patients in the pure group 1 day after surgery and the SR, OV20%, and OV9% values in the pure group 1 week after surgery were higher than those in the traditional group. These results suggested that the visual quality of patients in the pure group was better than that of patients in the traditional group during the early postoperative stage. Correspondingly, the UDVA and CDVA of the pure group 1 day after surgery were also higher than those of the traditional group. These differences were thought to be related to the higher IOP, the presence of residual OVDs, and the longer operation times in the traditional group, which caused AC inflammatory responses. No significant differences in objective visual quality indicators and subjective visual quality scale scores were observed between the two groups at 3 months after surgery, which indicated that both groups had good visual quality at 3 months after surgery.

The cornea is an important component of the refractive system of the eye. The maintenance of corneal thickness and transparency depends on the structural and functional integrity of corneal endothelial cells [33, 34]. The excessive loss of corneal endothelial cells can lead to the decompensation of corneal endothelial cell function, irreversible corneal oedema, and severe damage to visual function [35]. Corneal endothelial cells play decisive roles in the maintenance of normal physiological function in corneal tissues and are completely exposed to a nonphysiological environment during ICL implantation. Corneal endothelial cells can come into direct contact with perfusion fluid, viscoelastic agents, surgical instruments, and ICL, making them vulnerable to various factors. In previous EVO-ICL implantation studies using OVDs, Cao [36] reported a corneal endothelial cell loss rate of approximately 2% at 6 months after surgery. Lisa [37] reported a rate of 1.7% at 12 months after surgery. Pan [26] found that the rate was approximately 4.6% at 3 months after surgery. In this study, the loss rate of corneal endothelial cells in the pure group was approximately 2%, whereas that in the traditional group was approximately 2.3% at 3 months after surgery. No significant differences were observed before and after surgery for either group. The results showed that the pure group in this study was not associated with additional damage to corneal endothelial cells, with an outcome similar to the use of OVDs. The lack of additional corneal injury caused by continuous BSS perfusion in the pure group may be due to the shorter operation time in the pure group (2.897 ± 0.346 min), which was nearly half that of the traditional group. The AC remained stable during the operation, reducing the risk of mechanical injury due to the operation. In addition, the equilibrium liquefaction composition of BSS is similar to that of aqueous humour. BSS is noncytotoxic with mild properties and can maintain the pH and osmotic pressure of the AC. In addition, in this study, the BSS perfusion time was short, the flow rate was slow, and the perfusion pressure was appropriate. The continuous application of perfusion fluid could remove intraoperative exudates, tissue fragments, and blood clots, effectively reducing the risk of corneal endothelial cell injury and postoperative inflammatory reactions. In this study, no lesions of the transparent lens were observed in either group intraoperatively or 3 months after surgery, which indicated that pure ICL implantation could safely avoid the occurrence of OVD-related complications, such as corneal endothelial cell death and clear lens injury.

This study has some limitations. The observation time was short, and the sample size was relatively small. In the future, we will expand the sample size and extend the observation time.

## 5. Conclusions

In conclusion, the results of this study indicated that the pure ICL implantation method could avoid OVD-related IOP elevation, reducing the necessity and risk of IOP reduction management strategies, including the use of antiglaucoma drugs and AE discharge. Pure ICL is a safe and reliable method with clinical application that can reduce the postoperative inflammatory reaction, shorten the operation time, and reduce operating costs.

## Figures and Tables

**Table 1:** Preoperative demographics of the eyes undergoing the pure ICL implantation and traditional ICL implantation.

	Pure method	Traditional method	*P* value
Age (years)	24.650 ± 3.282 (21, 35 years)	24.750 ± 3.319 (21, 35 years)	*P*=0.893
Gender	Male : female = 8 : 12	Male : female = 10 : 10	
Spherical equivalent (D)	−11.306 ± 1.314 (−6.25∼−9.25)	−11.638 ± 1.431 (−6.25∼−9.50)	*P*=0.284
LogMAR UDVA	1.283 ± 0.226 (1.6–0.8)	1.230 ± 0.219 (1.6–0.8)	*P*=0.295
LogMAR CDVA	0.165 ± 0.103 (0.3–0)	0.182 ± 0.098 (0.3–0)	*P*=0.439

Note. *P*: *P* value of the data statistically compared between the pure ICL implantation group and the traditional ICL implantation groups. LogMAR: logarithm of the minimal angle of resolution; UDVA: uncorrected distance visual acuity; CDVA: corrected distance visual acuity; *D*: diopter.

**Table 2:** Time courses of the visual and refractive outcomes between the pure ICL implantation and the traditional ICL implantation groups.

	Preoperative period	Postoperative period			*P* value
		1 day	1 week	3 months	
LogMAR UDVA					
Pure method	1.283 ± 0.226	0.008 ± 0.066	−0.038 ± 0.067	−0.038 ± 0.067	*P*_0_ < 0.001^*∗*^
Traditional method	1.23 ± 0.219	0.07 ± 0.085	−0.025 ± 0.084	−0.042 ± 0.071	*P*_0_ < 0.001^*∗*^
*P* value	*P*_1_=0.295	*P*_1_ ≤ 0.001^*∗*^	*P*_1_=0.463	*P*_1_=0.747	

LogMAR BCVA					
Pure method	0.165 ± 0.103	−0.01 ± 0.071	−0.055 ± 0.064	−0.058 ± 0.071	*P*_0_ < 0.001^*∗*^
Traditional method	0.182 ± 0.098	0.048 ± 0.096	−0.03 ± 0.082	−0.058 ± 0.075	*P*_0_ < 0.001^*∗*^
*P* value	*P*_1_=0.439	*P*_1_=0.003^*∗*^	*P*_1_=0.133	*P*_1_=0.345	

Spherical equivalent (*D*)					
Pure method	−11.306 ± 1.314	−0.131 ± 0.253	−0.156 ± 0.202	−0.175 ± 0.206	*P*_0_ < 0.001^*∗*^
Traditional method	−11.638 ± 1.431	0.044 ± 0.259	−0.113 ± 0.16	−0.144 ± 0.169	*P*_0_ < 0.001^*∗*^
*P* value	*P*_1_=0.284	*P*_1_=0.003^*∗*^	*P*_1_=0.285	*P*_1_=0.460	

Note. *P*_0_: *P* value of the difference between the preoperative and three-month values; *P*_1_: *P* value of the difference between the pure ICL implantation and the traditional ICL implantation groups; ^*∗*^*P* < 0.05: significant difference. LogMAR: logarithm of the minimal angle of resolution; UDVA: uncorrected distance visual acuity; BCVA: best-corrected distance visual acuity; *D*: diopter.

**Table 3:** Time courses of the optical quality parameters after pure ICL implantation.

	Preoperative period	Postoperative period		
		1 day	1 week	3 months
MTF cut-off frequency (cpd)	48.48 ± 5.24	42.91 ± 6.15	45.32 ± 3.60	48.88 ± 3.92
*P* value		*P*_1_ < 0.001^*∗*^	*P*_2_=0.014^*∗*^	*P*_3_=0.999
SR	0.25 ± 0.06	0.23 ± 0.04	0.25 ± 0.06	0.26 ± 0.06
*P*value		*P*_1_=0.025^*∗*^	*P*_2_=0.951	*P*_3_=0.614
OV100%	1.63 ± 0.16	1.43 ± 0.20	1.52 ± 0.15	1.58 ± 0.16
*P*value		*P*_1_ < 0.001^*∗*^	*P*_2_=0.002^*∗*^	*P*_3_=0.167
OV20%	1.26 ± 0.20	1.10 ± 0.14	1.15 ± 0.23	1.27 ± 0.20
*P* value		*P*_1_ < 0.001^*∗*^	*P*_2_=0.147	*P*_3_=1.000
OV9%	0.71 ± 0.18	0.57 ± 0.12	0.70 ± 0.14	0.72 ± 0.12
*P* value		*P*_1_ < 0.001^*∗*^	*P*_2_=1.000	*P*_3_=1.000
OSI	0.41 ± 0.11	0.60 ± 0.16	0.57 ± 0.16	0.43 ± 0.11
*P* value		*P*_1_ < 0.001^*∗*^	*P*_2_=0.001^*∗*^	*P*_3_=0.945

Note. *P*_1_: *P* value of the difference in the visual quality parameters before surgery and one day after surgery; *P*_2_: *P* value of the difference in the visual quality parameters before surgery and one week after surgery; *P*_3_: *P* value of the difference in the visual quality parameters before surgery and three months after surgery. ^*∗*^*P* < 0.05: significant difference. MTF: modulation transfer function; OSI: objective scattering index; OV: OQAS value.

**Table 4:** Time courses of the optical quality parameters after traditional ICL implantation.

	Preoperative period	Postoperative period		
		1 day	1 week	3 months
MTF cut-off frequency (cpd)	47.82 ± 4.18	39.79 ± 4.78	43.83 ± 4.47	47.97 ± 3.93
*P* value		*P*_1_ < 0.001^*∗*^	*P*_2_ < 0.001^*∗*^	*P*_3_=0.880
SR	0.26 ± 0.05	0.21 ± 0.03	0.22 ± 0.03	0.27 ± 0.05
*P* value		*P*_1_ < 0.001^*∗*^	*P*_2_ < 0.001^*∗*^	*P*_3_=0.998
OV100%	1.62 ± 0.14	1.36 ± 0.26	1.44 ± 0.28	1.60 ± 0.15
*P* value		*P*_1_ < 0.001^*∗*^	*P*_2_=0.005^*∗*^	*P*_3_=0.987
OV20%	1.23 ± 0.26	1.01 ± 0.15	1.06 ± 0.15	1.18 ± 0.26
*P* value		*P*_1_ < 0.001^*∗*^	*P*_2_=0.003^*∗*^	*P*_3_=0.960
OV9%	0.72 ± 0.14	0.56 ± 0.08	0.62 ± 0.08	0.70 ± 0.17
*P* value		*P*_1_ < 0.001^*∗*^	*P*_2_ < 0.001^*∗*^	*P*_3_=0.989
OSI	0.41 ± 0.12	0.60 ± 0.19	0.55 ± 0.21	0.43 ± 0.11
*P* value		*P*_1_ < 0.001^*∗*^	*P*_2_=0.003^*∗*^	*P*_3_=0.992

Note. *P*_1_: *P* value of the difference in the visual quality parameters before surgery and one day after surgery; *P*_2_: *P*value of the difference in the visual quality parameters before surgery and one week after surgery; *P*_3_: *P* value of the difference in the visual quality parameters before surgery and three months after surgery. ^*∗*^*P* < 0.05: significant difference. MTF: modulation transfer function; OSI: objective scattering index; OV: OQAS value.

**Table 5:** Time courses of the visual quality indicators in the pure ICL implantation and traditional ICL implantation groups.

	Before operation		1 day after operation		1 week after operation		3 months after operation	
	Pure method	Traditional method	Pure method	Traditional method	Pure method	Traditional method	Pure method	Traditional method
MTF cut-off frequency	48.48 ± 5.24	47.82 ± 4.18	42.91 ± 6.15	39.79 ± 4.78	45.32 ± 3.60	43.83 ± 4.47	48.88 ± 3.92	47.97 ± 3.93
*P* value	*P*=0.533		*P*=0.013^*∗*^		*P*=0.105		*P*=0.301	
SR	0.25 ± 0.06	0.26 ± 0.05	0.23 ± 0.04	0.21 ± 0.03	0.25 ± 0.06	0.22 ± 0.03	0.26 ± 0.06	0.27 ± 0.05
*P* value	*P*=0.614		*P*=0.009^*∗*^		*P*=0.003^*∗*^		*P*=0.707	
OV100%	1.63 ± 0.16	1.62 ± 0.14	1.43 ± 0.20	1.36 ± 0.26	1.52 ± 0.15	1.44 ± 0.28	1.58 ± 0.16	1.60 ± 0.15
*P* value	*P*=0.653		*P*=0.161		*P*=0.149		*P*=0.655	
OV20%	1.26 ± 0.20	1.23 ± 0.26	1.10 ± 0.14	1.01 ± 0.15	1.15 ± 0.23	1.06 ± 0.15	1.27 ± 0.20	1.18 ± 0.26
*P* value	*P*=0.619		*P*=0.004^*∗*^		*P*=0.047^*∗*^		*P*=0.116	
OV9%	0.71 ± 0.18	0.72 ± 0.14	0.57 ± 0.12	0.56 ± 0.08	0.70 ± 0.14	0.62 ± 0.08	0.72 ± 0.12	0.70 ± 0.17
*P* value	*P*=0.689		*P*=0.577		*P*=0.002^*∗*^		*P*=0.630	
OSI	0.41 ± 0.11	0.41 ± 0.12	0.60 ± 0.16	0.60 ± 0.19	0.57 ± 0.16	0.55 ± 0.21	0.43 ± 0.11	0.43 ± 0.11
*P* value	*P*=0.850		*P*=0.932		*P*=0.762		*P*=0.954	

Note. *P*: *P* value of the difference between visual quality indicators in the pure ICL implantation and traditional ICL implantation groups; ^*∗*^*P* < 0.05: significant difference; MTF: modulation transfer function; OSI: objective scattering index; OV = OQAS value.

**Table 6:** NEI-RQL-42 scores before and 3 months after surgery in the pure ICL implantation and traditional ICL implantation groups.

	Pure method			Traditional method		
	Pre	3 mo post	*P* value	Pre	3 mo post	*P* value
Total score	60.568 ± 3.772	83.685 ± 2.675	<0.001^*∗*^	60.495 ± 3.866	83.493 ± 2.663	<0.001^*∗*^
Clarity of vision	64.327 ± 2.894	87.045 ± 3.184	<0.001^*∗*^	64.422 ± 2.825	86.284 ± 3.551	<0.001^*∗*^
Expectation	30.500 ± 3.558	81.436 ± 3.721	<0.001^*∗*^	30.555 ± 3.561	80.143 ± 4.276	<0.001^*∗*^
Near vision	81.508 ± 3.361	81.217 ± 3.448	0.714	82.244 ± 3.383	81.722 ± 3.404	0.556
Far vision	81.920 ± 3.687	83.720 ± 3.105	0.033^*∗*^	81.925 ± 3.751	83.666 ± 3.231	0.026^*∗*^
Visual fatigue	74.548 ± 2.447	77.835 ± 2.589	<0.001^*∗*^	74.488 ± 2.407	77.580 ± 2.824	<0.001^*∗*^
Activity limitations	40.516 ± 3.346	87.687 ± 2.288	<0.001^*∗*^	40.463 ± 3.250	87.210 ± 3.065	<0.001^*∗*^
Glare	73.504 ± 3.083	70.596 ± 3.300	0.001^*∗*^	73.548 ± 3.136	70.509 ± 3.337	<0.001^*∗*^
Symptoms	73.155 ± 3.114	78.344 ± 2.407	<0.001^*∗*^	72.491 ± 2.859	78.413 ± 2.092	<0.001^*∗*^
Dependence on correction	28.493 ± 1.818	98.288 ± 0.952	<0.001^*∗*^	28.528 ± 1.782	98.454 ± 0.916	<0.001^*∗*^
Worry	49.473 ± 2.213	75.534 ± 3.335	<0.001^*∗*^	49.704 ± 2.152	75.643 ± 3.284	<0.001^*∗*^
Suboptimal correction	74.518 ± 3.777	89.625 ± 3.951	<0.001^*∗*^	74.072 ± 3.870	89.40]7 ± 4.006	<0.001^*∗*^
Appearance	48.555 ± 3.042	90.819 ± 3.666	<0.001^*∗*^	49.154 ± 2.878	90.475 ± 3.415	<0.001^*∗*^
Satisfaction with correction	58.498 ± 3.490	90.211 ± 3.704	<0.001^*∗*^	57.490 ± 3.387	89.448 ± 3.383	<0.001^*∗*^

Note. *P*: *P* value of NEI-RQL-42 scores statistically compared before and 3 months after surgery in the pure ICL implantation and traditional ICL implantation groups; ^*∗*^*P* < 0.05: the difference is statistically significant.

**Table 7:** NEI-RQL-42 scores before and 3 months after surgery in the pure ICL implantation and traditional ICL implantation groups.

	Pre			3 mo post		
	Pure method	Traditional method	*P* value	Pure method	Traditional method	*P* value
Total score	60.568 ± 3.772	60.495 ± 3.866	0.932	83.685 ± 2.675	83.493 ± 2.663	0.748
Clarity of vision	64.327 ± 2.894	64.422 ± 2.825	0.926	87.045 ± 3.184	86.284 ± 3.551	0.316
Expectation	30.500 ± 3.558	30.555 ± 3.561	0.946	81.436 ± 3.721	80.143 ± 4.276	0.153
Near vision	81.508 ± 3.361	82.244 ± 3.383	0.332	81.217 ± 3.448	81.722 ± 3.404	0.512
Far vision	81.920 ± 3.687	81.925 ± 3.751	0.995	83.720 ± 3.105	83.666 ± 3.231	0.939
Visual fatigue	74.548 ± 2.447	74.488 ± 2.407	0.912	77.835 ± 2.589	77.580 ± 2.824	0.675
Activity limitations	40.516 ± 3.346	40.463 ± 3.250	0.943	87.687 ± 2.288	87.210 ± 3.065	0.433
Glare	73.504 ± 3.083	73.548 ± 3.136	0.950	70.596 ± 3.300	70.509 ± 3.337	0.907
Symptoms	73.155 ± 3.114	72.491 ± 2.859	0.324	78.344 ± 2.407	78.413 ± 2.092	0.891
Dependence on correction	28.493 ± 1.818	28.528 ± 1.782	0.931	98.288 ± 0.952	98.454 ± 0.916	0.429
Worry	49.473 ± 2.213	49.704 ± 2.152	0.637	75.534 ± 3.335	75.643 ± 3.284	0.883
Suboptimal correction	74.518 ± 3.777	74.072 ± 3.870	0.603	89.625 ± 3.951	89.407 ± 4.006	0.807
Appearance	48.555 ± 3.042	49.154 ± 2.878	0.368	90.819 ± 3.666	90.475 ± 3.415	0.665
Satisfaction with correction	58.498 ± 3.490	57.490 ± 3.387	0.194	90.211 ± 3.704	89.448 ± 3.383	0.339

Note. *P* value: NEI-RQL-42 scores before and 3 months after surgery statistically compared between the pure ICL implantation and the traditional ICL implantation groups.

**Table 8:** Comparison of corneal endothelial cell density within and between the pure ICL implantation and the traditional ICL implantation groups.

	Before operation	3 months after operation
Pure method	2717.280 ± 133.619/mm^2^	2663.650 ± 129.517/mm^2^
Traditional method	2702.380 ± 158.959/mm^2^	2639.600 ± 141.652/mm^2^

Note. Comparisons of ECD within and between the pure ICL implantation and traditional ICL implantation groups were all *P* > 0.05.

## Data Availability

The datasets used and/or analysed during the current study are available from the corresponding author on reasonable request.

## Abbreviations

ICL: Intraocular collamer lens
OV: OQAS II values
OSI: Objective scatter index
MTF: Modulation transfer function
UDVA: Uncorrected distance visual acuity
BCVA: Best-corrected distance visual acuity.
